# Veterans Affairs Providers' Beliefs About the Contributors to and Responsibility for Reducing Racial and Ethnic Health Care Disparities

**DOI:** 10.1089/heq.2019.0018

**Published:** 2019-08-23

**Authors:** Johanne Eliacin, Brooke Cunningham, Melissa R. Partin, Amy Gravely, Brent C. Taylor, Howard S. Gordon, Somnath Saha, Diana J. Burgess

**Affiliations:** ^1^ Center for Health information and Communication, CHIC, Health Services Research & Development, Richard L. Roudebush VA Medical Center, Indianapolis, Indiana.; ^2^ Department of Psychology, Indiana University Purdue University at Indianapolis, Indianapolis, Indiana.; ^3^ Health Services Research, Regenstrief Institute, Inc., Indianapolis, Indiana.; ^4^ ACT Center of Indiana, Indianapolis, Indiana.; ^5^ Department of Family Medicine and Community Health, Minneapolis, Minnesota.; ^6^ Center for Chronic Disease Outcomes Research (a VA HSR&D Center of Excellence), Veterans Affairs Medical Center, Minneapolis, Minnesota.; ^7^ Department of Medicine, University of Minnesota, Minneapolis, Minnesota.; ^8^ Jesse Brown Veterans Affairs Medical Center and Center of Innovation for Complex Chronic Healthcare, Chicago, Illinois.; ^9^ Section of Academic Internal Medicine and Geriatrics, Department of Medicine, University of Illinois at Chicago College of Medicine, Chicago, Illinois.; ^10^ Section of General Internal Medicine, VA Portland Health Care System, Portland, Oregon.; ^11^ Division of General Internal Medicine and Geriatrics, Oregon Health & Science University, Portland, Oregon.

**Keywords:** health care disparities, providers, minority patients, veterans

## Abstract

**Purpose:** Providers' beliefs about the causes of disparities and the entities responsible for addressing these disparities are important in designing disparity-reduction interventions aimed at providers. This secondary analysis of a larger study is aimed at evaluating perceptions of providers regarding the underlying causes of racial health care disparities and their views of who is responsible for reducing them.

**Methods:** We surveyed 232 providers at 3 Veterans Affairs (VA) Medical Centers.

**Results:** Sixty-nine percent of participants believed that minority patients in the United States receive lower quality health care. Most participants (64%) attributed differences in quality of care for minority patients in the VA health care system primarily to patients' socioeconomic status, followed by patient behavior (43%) and provider behaviors (33%). In contrast, most participants believed that the VA and other health care organizations (75%) and providers (70%) bear the responsibility for reducing disparities, while less than half (45%) believed that patients were responsible. Among provider-level contributors to disparities, providers' poor communication was the most widely endorsed (48%), while differences in prescribing of medications (13%) and in provision of specialty referrals (12%) were the least endorsed.

**Conclusions:** Although most providers in the study did not believe that providers contribute to disparities, they do believe that they, along with health care organizations, have the responsibility to help reduce them. Interventions might focus on directly offering providers concrete ways that they can help reduce disparities, rather than focusing on simply raising awareness about disparities and their contributions to them.

## Introduction

Since the publication of the seminal Institute of Medicine report on racial and ethnic disparities in health care in 2002,^1^ numerous studies have presented robust evidence demonstrating health care disparities in the U.S. health care system.^2–5^ Although the factors contributing to health care disparities are complex, involving both patient and provider factors, as well as broader societal and organizational contexts,^1^ there is consensus from policy makers and health care leaders that health care providers play a critical role in eliminating health care disparities.^6–8^

In response, there has been a proliferation of disparity-reduction training activities aimed at providers.^9–11^ Yet, there is scant evidence that such efforts have been effective.^12–14^ There is also limited research on how to effectively communicate with providers about health care disparities to engage them in disparity reduction efforts.^15,16^ This is a major barrier to the development of effective health care disparity interventions, as communication that contradicts people's preexisting beliefs can lead to resistance^17^ and “boomerang effects,” in which the communication has the opposite of its intended effects.^18^ Understanding how to effectively communicate with providers about health care disparities and motivating them to participate in health disparity intervention are essential given their significant role in health care disparities and the limited effectiveness of existing educational trainings in improving providers' views of minority groups and health care disparities.^10,13,14^

Although many providers believe that health care disparities exist, they also tend to view these disparities as unlikely to occur in their individual practices.^19–23^ Moreover, providers are less likely to endorse provider behaviors as causes for disparities despite evidence pointing to their contributory role.^21,23,24^ They tend to downplay their own personal biases and attribute health care disparities to others' actions and characteristics such as patients' health behaviors.^21,23^ For example, even providers who agreed that racial minority patients receive different health care treatments, such as dissimilar diagnoses and procedures compared to White patients, endorsed patient behaviors as the primary explanation for health care disparities over provider or system factors.^22^ In fact, this phenomenon has been consistently reported in several studies over the past decade^22,23,25^ and appears to persist among new medical residents despite increased attention to disparities in medical education.^20^

The present study is part of a larger project aimed at developing effective communication strategies to engage providers in disparity-reduction activities, using a message-matching strategy that tailors messages based on providers' preexisting beliefs.^15,16,26^ In this secondary analysis, our primary objectives are to examine the beliefs of health care providers about the contribution of health care disparities at a global and granular level, and about responsibility for reducing health care disparities, to inform future disparity-reduction interventions and engagement strategies targeted at providers.

We focus on describing providers' global beliefs about the causes of health care disparities (e.g., provider and patient contribution), as well as more granular beliefs (e.g., provider bias; patient preferences for certain types of treatment). We also assess beliefs about responsibility for addressing disparities. While it is assumed that fostering providers' awareness of health care disparities is necessary for motivating providers to engage in disparities reducing efforts and improving public health,^15,27,28^ it may be possible to engage providers more directly, without targeting their biases and underlying beliefs about health care disparities. For example, such interventions may involve promoting changes in clinical practice or using better models of individualized care that includes utilizing patients' cultural backgrounds to encourage healthy behaviors.^28^

### Context

The present project focused on providers working in the Veterans Affairs Healthcare Systems (VAHCSs) because of its growing racial minority veteran population, its leading role in training health professionals, and its recent initiatives aimed at improving health equity for veterans. The number of racial and ethnic minority veterans has rapidly increased over the last 30 years. In 2014, over 5 million veterans, about 23% of the veteran population, were of racial minority background, and it is estimated that this number will increase to 36% by 2040.^29^ Second, the VAHCS is the largest educator and trainer of health professionals in the United States. Over 65% of all U.S. trained physicians and nearly 70% of Veterans Affairs (VA) physicians have had VA training before employment.^30^ As such, the VA plays a significant role in shaping the training, attitudes, and behaviors of the country's future health care workforce. Thus, assessing VA providers' views of health care disparities is a crucial first step to understand the culture of health equity promotion in the VAHCS. Third, the VAHCS provides a unique opportunity to study health care disparity efforts given that it is the largest integrated health care system in the United States, serving more than 8.9 million Veterans each year.^30^ Furthermore, in recent years, the VA has renewed its efforts to eliminate health care disparities and has invested in many quality improvement initiatives, such as improvement in race data collection and educational initiatives directed at providers and policy makers.^31,32^

Despite the VAHCSs leading role in health care education and health equity promotion, to date, there is a dearth of evidence of providers' views of health care disparities in the VAHCS (e.g., whether disparities exist and its underlying causes). This study addresses this specific gap and further advances existing research on providers' perspectives about health care disparities by assessing providers' views on who has the responsibility for addressing health care disparities. It also examines the linkage between providers' beliefs about disparities and their views on who should lead efforts to eliminate them, which may bring new and valuable insights to inform organizational intervention strategies to promote health equity.

## Methods

This study is part of a larger sequential mixed methods study, Enhancing Motivation of Providers On Work to Eliminate Racial disparities (EMPOWER), which was conducted at three VA Medical Centers.^15,16^ The primary objective of EMPOWER was to understand how to effectively communicate with health care professionals about health care disparities. It involved two phases. Phase one included assessment of providers' views of and attributions to health care disparities, and phase two focused on (1) a factorial experiment that tested the impact of tailored narratives on identifying and increasing providers' readiness to act to reduce disparities, and (2) identification of narrative type that leads to the highest level of participation in disparity-reduction training among providers. The data presented in this part are part of the first phase of the parent study.

### Survey design, measurements, and variables

We administered a survey designed for this study to assess provider's beliefs about health care for culturally and ethnically diverse patients. In the absence of existing validated surveys on this topic, ours was constructed based on a literature review of factors contributing to differences in health care quality for racial and ethnic minority patients. It consisted of single items that asked participants about their beliefs about health care and other broad issues. See Appendix A1 for a copy of the survey and Appendix A2 for information sheet.

The following item was used to assess providers' perceptions of disparities: “Please indicate how much you agree or disagree with the following statement (1) minority patients in the U.S. as a whole receive lower quality health care than White patients.” Responses to survey questions ranged from strongly agree to strongly disagree on a seven-point Likert scale. The seven-point Likert scale gave responders a broader range of options and facilitated completion of the survey. However, for parsimony and ease of interpretation, we converted the seven-point response range to a three-point measure (agree, neutral, disagree) by grouping similar responses together. Comparisons of findings from the two measures yielded similar results.

Moreover, we assessed providers' beliefs of the extent to which (1) patient behavior, (2) provider behavior, and (3) the social and economic conditions in which patients live contribute to racial differences in health care quality, with responses ranging from 0=not at all to 6=a great deal. We examined these attributional categories by asking participants to identify how specific patient, provider, and organizational factors such as providers' biases and patients' health behaviors contribute to differences in health care for minority patients. We also evaluated providers' beliefs about what degree of responsibility (1) VA patients, (2) VA providers, and (3) VA health care system should have for reducing disparities. Reponses to these questions ranged from 0=not at all to 6=a great deal, which were reduced to not at all, neutral, or a great deal. We compared findings from the seven-point scale to the three-point scale and there were no major differences. We examined the distribution of provider characteristics, and beliefs about disparities, and used multivariate logistic regression to examine the association between provider characteristics and beliefs about disparities. We used bootstrapping methods to compare the percentages statistically, obtaining 95% confidence intervals.^33^ We used SAS version 9.2.

### Sample and recruitment procedures

The study was conducted at two large Midwestern VA Medical Centers and one VA Medical Center in the Southern region of the United States. These sites were purposefully selected because of different population compositions (mostly African American at the Southern sites and predominantly White patients at the Midwestern sites), as well as differential racial attitudes of people living in the Midwestern and Southern regions of the United States.^34,35^ Moreover, previous research has shown that clinical practices with varied proportions of minority patients have different workplace environments, provider characteristics and experiences, and patient care quality.^36–38^ Therefore, we wanted to include in our sample a broad range of providers from different VA facilities that have low and high proportions of minority patients to facilitate maximum variations in participants' demographic characteristics, racial attitudes, and experiences with patients of minority backgrounds.

Participants in the study consisted of nurse practitioners, physicians, and physician assistants. Recruitment methods are described in detail elsewhere.^15,26^ Initially, we recruited participants through email with postal mail follow-up. We obtained a list of all physicians, nurse practitioners, and physician assistants from the three facilities from the Veterans Health Administration (VHA) intranet (*N*=637). Given the low response rates, we changed our recruitment approach to include in-person recruitment, in which the investigator at each site provided information about the study at staff meetings and invited providers to complete the survey, on paper or online. Overall, between January 6, 2014, and December 8, 2014, 273 participants completed the survey online. Of these, 134 participants completed the survey online, and 139 completed a paper survey. The response rate for one site was 59%. The response rates for the other two sites were underestimated (31% and 27%) because we were not able to track the surveys that were not deliverable. All surveys were self-administered and took 10–15 min to complete.

## Results

We received responses from 273 participants. Due to missing data, only 232 participants were included in the final analyses. Most participants were physicians and predominantly White. The sample was equally divided by gender. Half of the participants reported that they had cultural competency training and nearly half that practiced in clinical settings that they estimated had greater than 25% racial and ethnic minority patients. Additional information on provider demographic and practice characteristics is presented in Table 1.

**Table 1.** Provider Characteristics (%)

Variable	Total, *N*=232
Gender	
M	50.2
F	49.8
Race	
White	80.4
Black	2.6
Latino	3.1
Native American	0.9
Asian	11.4
Other	1.3
Decline	0.4
Age	
<45	27.9
45–59	42.0
60+	30.1
Site	
Charleston	18.6
Chicago	22.0
Minneapolis	59.5
Had cultural competency training	
No	49.1
Yes	50.9
Professional status	
NP	22.0
MD	69.0
PA	7.3
Other	1.7
Percentage of patients that are non-White	
Less than 10% NW	15.4
10–25% NW	38.9
Greater than 25% NW	45.7

NW, non-White.

As shown in Table 2, most participants, 69%, reported that minority patients in the United States receive lower quality care. Sixty-three percent of participants attributed health care disparities to patients' socioeconomic status (SES) conditions, 42.8% attributed disparities to patient behavior, and 33.2% attributed disparities to provider behavior.

**Table 2.** Provider Beliefs: Contributors to and Responsibility for Reducing Disparities

	Percentage endorsing						
	Strongly, somewhat, slightly disagree			Neither agree nor disagree	Strongly, somewhat, slightly agree		
Minority patients in the United States as a whole receive lower quality health care than White patients	25.11			5.53	69.36		

	Not at all				A great deal		
	0	1	2	3	4	5	6
How much does SES conditions in which the patient lives contribute to racial differences in health care quality?	22.03			14.41	63.56		
How much does patient behavior contribute to racial differences in health care quality?	36.86			20.34	42.80		
How much does provider behavior contribute to racial differences in health care quality?	40.00			26.81	33.19		
Contributors to disparities: specific items							
Patient factors							
Patient health behaviors (diet, exercise, adherence)	20.69			21.98	57.33		
Patient mistrust in the medical system	23.18			21.46	55.36		
Patient misunderstanding of treatment	26.72			20.69	52.59		
Patient attitudes/beliefs about provider	30.17			20.69	49.14		
Patient preferences for type of treatment	35.22			25.22	39.57		
Patient uncooperativeness	37.23			16.02	46.75		
Poor communication by patients	39.22			23.28	37.5		
Patient lack of effort	44.21			21.03	34.76		
Lack of motivation on the part of patients	41.63			22.32	36.05		
Provider factors							
Poor communication by providers	32.76			18.97	48.28		
Provider attitudes/beliefs about minority patients	41.03			21.37	37.61		
Provider biases in decision making	50.64			22.32	27.04		
Differences in prescribing of medications	66.38			20.26	13.36		
Difference in provision of specialty referrals	67.97			20.35	11.69		
Organizational factors							
Lack of time/resources for providers to address social issues	22.32			17.6	60.09		
Provider workforce diversity	49.79			22.32	27.90		
Difference in provision of specialty referrals	67.97			20.35	11.69		
Responsibility for reducing disparities							
VA health care system responsible for reducing racial health care disparities	12.82			11.97	75.21		
VA providers responsible for reducing racial health care disparities	14.1			16.24	69.66		
VA patients responsible for reducing racial health care disparities	33.48			21.46	45.06		

SES, socioeconomic status; VA, Veterans Affairs.

We then examined specific contributors to disparities within three attributional categories as follows: patient factors, provider factors, and organizational factors. Among patient factors, participants identified several patient behaviors as contributors to health care disparities such as patient uncooperativeness (47%), patient attitudes/beliefs about provider (49%), patient misunderstanding of treatment (53%), and patient health behaviors (57%). Poor communication by providers was identified by almost half of the participants (48%) as a major contributor to health care inequality. For organizational-level factors, few participants (27%) viewed lack of provider workforce diversity as a contributor. By contrast, many viewed lack of time/resource for providers to address social issues (60%) as a contributor.

In terms of who the participants viewed as being responsible for reducing disparities, 75% of the participants identified the VA and other health care organizations as having responsibility, followed by providers (70%) and then patients (45%). Multivariate analyses did not reveal significant associations between participants' views on the existence of health care disparities and providers' characteristics, site, professional status, or training in cultural competence.

## Discussion

Our goal in this study was to describe VA health care providers' beliefs about the contributors to and responsibility for reducing racial and ethnic health care disparities. To our knowledge, our study is the first to assess beliefs about responsibility, as well as contributors, to disparities. In so doing, we sought to set the stage for future studies to help develop engagement and intervention strategies that would be effective with providers, based on their preexisting beliefs. Based on provider surveys from three VAHCSs from the Midwestern and Southern regions in the United States, we found that most participants recognized the existence of health care disparities in the U.S. health care system. Consistent with previous findings,^21,23,25^ providers in this study were more likely to identify patient-related factors over provider-related factors as contributors to differences in health care quality for minority patients. In addition, there was a great deal of variability in providers' responses in terms of the specific contributors endorsed, at the patient, provider, and organizational levels. For example, although few participants endorsed provider differences in prescribing of medications (13%) and in provision of specialty referrals (12%) as contributing to disparities, many more endorsed providers' poor communication (48%).

Despite the fact that most participants did not believe that providers contribute to disparities, the majority did believe that providers and health care organizations are responsible for reducing them. The idea that providers who are resistant to the notion that they contribute to health care disparities might nonetheless be motivated to address disparities in their own practice was a central premise of the EMPOWER project, which sought to develop narratives (or stories) to engage such resistant providers. In our qualitative and quantitative studies,^15,16,26^ we found that providers who did not believe providers contributed to disparities responded more positively to “provider success” narratives, in which the provider in the story successfully resolved a problem involving Black patient compared with “Provider Bias” narratives in which a Black patient experienced racial bias by a provider.^15,16^ These findings, along with the results of our secondary analysis, underscore the need to carefully consider how activities aimed at reducing racial health care disparities are framed.

Interventions might focus on directly offering providers concrete ways that they and their organization can help reduce health care disparities and improve patient care (such as improving patient–provider communication), rather than focusing squarely on the issues of racial bias. In contrast, it could be argued that interventions that bypass systemic racism inherent in health care and the larger society^39,40^ reinforce an individualistic ideology, dominant in the United States, in which individuals (e.g., patients) are responsible for fixing their own problems.^41^ In the short run, however, it may be more effective for health care organizations to understand what values and motivations held by providers, such as their feelings of responsibility for addressing disparities, will be most fruitful to target, if real change is to occur.

In addition, departing from prior research that assessed providers' beliefs of the existence of and contributors to health care disparities,^23,25^ this study included items such as patients' SES and social conditions of life, which are broadly considered as social determinants of health.^42,43^ Participants viewed the social contexts of patients' lives as influential in patients' health care experiences and outcomes.

In most studies on providers' perceptions of health care disparities, much of the discussion on who is responsible for health care disparities has focused on the individual—either the individual patient or provider—and the belief that individual health behaviors are the most important determinants of health.^44–46^ However, recent approaches to health care disparities have moved beyond individual responsibility to embrace a social determinant perspective.^47,48^ This theoretical perspective sees organizational contexts and structures, sociohistorical factors, inequalities, and political ideologies as influencing health behaviors.^49,50^ Our finding demonstrating providers' support for a greater role in addressing patients' social contexts addresses a gap in perceptions of disparities in the literature and suggests the need for future studies to integrate in their surveys questions about system factors and perceptions of responsibility for addressing disparities.

These findings have implications for VAHCS health equity initiatives. Specifically, our findings suggest that system-level health care disparity reduction efforts rather than individual behavioral changes might be more effective and desirable. They provide evidence to support and encourage the development of programs that seek to address social determinants of health as contributors to veterans' health. The VAHCS' current “Whole Health” initiative is an example of such programs.^51^ It seeks to provide person-centered care that takes into consideration social aspects of patients' lives in their care—their values, needs, and goals. Specifically, it encourages patients and providers to develop partnerships to identify what matters most to patients and to help empower and equip patients to meet their personalized goals. Building on this Whole Health approach and findings from this study, future interventions may address what resources and organizational support providers may need to effectively provide “whole healthcare” to patients that go beyond their presenting health problems and to be able to address social contexts that impact their health and well-being.

Our study participants downplayed the importance of workforce diversity for reducing disparities. In contrast, research suggests that a diverse workforce is a key factor in reducing disparities.^1,52–56^ This is an area for future intervention at the organizational level, especially in the VA where the minority Veteran population is rapidly increasing. It will be important to make providers more aware of the importance of workforce diversity. Future studies on patients' views of workforce diversity are also needed.

Participants acknowledged that ineffective patient–provider communication contributes to health inequality. This finding is consistent with previous studies.^57^ Shared decision-making, an effective communicative approach, presents opportunities to deliver patient-centered care and to address sociocultural contexts of patients' lives, as well as issues related to social determinants of health.^58–61^ Future steps in improving health care quality and equity for minority patients could focus on increasing providers and patients' participations in shared decision-making processes.

Our study provides new data on providers' views of health care disparities and factors that contribute to these disparities. Yet, several limitations should be noted. We constructed a survey based on the literature to assess providers' perspectives on health care disparities. Our findings, although not based on validated measures, provide a preliminary assessment of VA providers' views on health care disparities. We also condensed the survey from a seven-point Likert scale to a three-point scale, which may have affected the reliability of our measure and data interpretation. Although our findings are descriptive at this stage, they are the first steps in generating hypotheses for understanding providers' causal attributions of health care disparities, mapping progress of health equity efforts in terms of changes in providers' attitudes and beliefs, and for developing valid measures for future studies. Moreover, it is possible that our relatively low response rate could have resulted in a sample in which providers who participated in the study were more aware about issues of disparities and more motivated to address it. Our response rate and limited sample size may also account for the lack of identified relationships between site, participants' views of racial health care disparities and providers' characteristics, site, and professional status.

Providers in this study were all VHA providers. While racial disparities have been documented in many areas of VHA care and continue to persist, the VHA has invested in a wide range of efforts to eliminate them and has met with some success.^31,62,63^ Our sample also may not be representative of all VA health care providers and did not facilitate full evaluation of providers' characteristics across multiple factors. Our sample also consisted of providers who are primarily from large urban areas with a growing minority population. Therefore, our findings may not be generalizable to the broader U.S. health care system.

## Conclusion

This study offers insights into how to develop strategies for engaging providers in disparity-reduction activities. Despite the fact that most providers in our study did not believe providers contributed to disparities, they did believe that they and health care organizations were responsible for addressing them. They also believe that more efforts are needed to address social determinants of health to successfully address health care disparities. Health care leaders, policy makers, and researchers should take advantage of this receptivity to develop, implement, and test initiatives that address health care system and social determinants of health care disparities.

## Patient Anonymity and Informed Consent

This study was approved by the VA Central Institutional Review Board (IRB) on January 22, 2013 (C-IRB 12-28). The interviewer obtained written informed consent prior to the interview.

## Abbreviations Used

EMPOWER: Enhancing Motivation of Providers On Work to Eliminate Racial disparities
SES: socioeconomic status
VA: Veterans Affairs
VAHCS: Veterans Affairs Healthcare System

## Appendix A1. Provider Perspectives on Delivering Care to Diverse Patients Survey

A. **It has been documented that minority patients in the Veterans Affairs, on average, receive lower quality health care than White patients.**
  1. In your opinion, how much does each of the following factors contribute to these racial differences in health care quality:
    **Table:**
    	**Not at all**						**A great deal**
    	**1**	**2**	**3**	**4**	**5**	**6**	**7**
    a. Patient behavior	□	□	□	□	□	□	□
    b. Provider behavior	□	□	□	□	□	□	□
    c. The social and economic conditions in which patients live	□	□	□	□	□	□	□
  2. In your opinion, how much should each of the following groups be responsible for reducing these racial differences in health care quality?
    **Table:**
    	**Not at all**						**A great deal**
    	**1**	**2**	**3**	**4**	**5**	**6**	**7**
    a. VA patients	□	□	□	□	□	□	□
    b. VA health care providers	□	□	□	□	□	□	□
    c. The VA health care system	□	□	□	□	□	□	□
  3. How much do you think that each of the following factors contributes to racial and ethnic differences in the quality of care of clinically similar patients?
    **Table:**
    	**Not at all**						**A great deal**
    	**1**	**2**	**3**	**4**	**5**	**6**	**7**
    a. Patient uncooperativeness	□	□	□	□	□	□	□
    b. Differences in provision of specialty referrals	□	□	□	□	□	□	□
    c. Provider work force diversity	□	□	□	□	□	□	□
    d. Patient attitudes/beliefs about provider	□	□	□	□	□	□	□
    e. Patients' lack of effort	□	□	□	□	□	□	□
    f. Patient preference for type of treatment	□	□	□	□	□	□	□
    g. Provider attitudes/beliefs about minority patients	□	□	□	□	□	□	□
    h. Patient understanding of treatment	□	□	□	□	□	□	□
    i. Lack of time/resources for providers to address social issues	□	□	□	□	□	□	□
    j. General miscommunication with patients by providers	□	□	□	□	□	□	□
    k. Patient health behaviors (diet, exercise, adherence)	□	□	□	□	□	□	□
    l. Patient mistrust in the medical system	□	□	□	□	□	□	□
    m. Patients' social and economic circumstances	□	□	□	□	□	□	□
    n. Differences in prescribing of medications	□	□	□	□	□	□	□
    o. Lack of motivation on the part of patients	□	□	□	□	□	□	□
  4. Please indicate how much you agree or disagree with the following questions:
    **Table:**
    	**Strongly disagree**	**Somewhat disagree**	**Slightly disagree**	**Slightly agree**	**Somewhat agree**	**Strongly agree**
    a. Minority patients in the United States as a whole receive lower quality health care than White patients	□	□	□	□	□	□
    b. The VA health care system treats people unfairly based on their race and ethnicity	□	□	□	□	□	□
  5. Please indicate how much you agree or disagree with the following questions:
    **Table:**
    	**Strongly disagree**	**Somewhat disagree**	**Slightly disagree**	**Slightly agree**	**Somewhat agree**	**Strongly agree**
    a. Conscientious patients deserve better health care than those with self-inflicted problems	□	□	□	□	□	□
    b. Those who contribute the most to society should get better health care	□	□	□	□	□	□
    c. More health care dollars should be spent on those that contribute most to society	□	□	□	□	□	□
    d. Compliant patients are entitled to more of my time than noncompliant ones	□	□	□	□	□	□
B. **The following are some questions about you and your practice. Please answer the questions to the best of your ability.**
  1. What is your gender? □ Male □ Female
  2. Which of the following best describes your racial or ethnic background? (Please check all that apply):
    □ African American/American-born Black
    □ African-born Black
    □ Asian or Pacific Islander
    □ Latino or Latin American or Hispanic
    □ Native American/Alaska Native
    □ White/European American
    □ Other: __________________________________________
  3. Were you born in the United States?
    □ Yes
    □ No
  4. Did you attend medical school in the United States?
    □ Yes
    □ No
  5. Please indicate your current professional status (select one or more):
    □ Nurse Practitioner
    □ Physician (MD, DO)
    □ Physician Assistant
    □ Other:_______________________________________________
  6. Approximately what percent of your patients are non-White: ______________%
  7. Have you ever participated in cultural competency training or training related to health care disparities?
    □ Yes **If Yes**, approximately how many hours of training did you engage in: _____________
    □ No
  8. How helpful did you think your cultural competency training was?
    **Table:**
    **Not at all**						**A great deal**
    **1**	**2**	**3**	**4**	**5**	**6**	**7**
    □	□	□	□	□	□	□
C. **Would you be interested in and willing to participate in a 60 min interview (at a time that is convenient for you), to provide input on developing educational messages to improve the care of diverse patients?**
  □ Yes
  □ No
  ***Thank you so much for your participation. Please contact the Principal Investigator, Dr. Diana***
  ***J. Burgess (diana.burgess@va.gov; 612-467-1591) if you have any questions or concerns.***

## Appendix A2. Information Sheet 1 for Phase 1 Screening Survey “Provider Perspectives on Delivering Care to Diverse Patients”

1. **Introduction:** You are being invited to take part in a research study that is being funded by the Department of Veterans Affairs. Before you decide to take part, it is important for you to know why the research is being done and what it will involve. This includes any potential risks to you, as well as any potential benefits you might receive.
  Read the information below closely and discuss it with family and friends if you wish. Ask one of the study staff if there is anything that is not clear or if you would like more details (see List no. 10 for study staff). If you do decide to take part, you will be asked to click a box to indicate that you received all of the information below
2. **Background and purpose:**
  a. This survey is part of a research project funded by VA Health Services Research and Development, in partnership with a number of Veterans Affairs (VA) organizations, including the National Center for Health Promotion, the Office of Patient Centered Care and Cultural Transformation, the VA Centers of Excellence in Primary Care Education, and the VHA Employee Education System. We hope this project will help us improve the delivery of health care in the VA for culturally and ethnically diverse patients.
  b. The purpose of this study is to develop educational initiatives for providers that align with their beliefs about health care for culturally and ethnically diverse patients, to improve the delivery of health care in the VA for these patients.
  c. You are being invited to participate in this study because you are a clinical provider at your VA facility.
3. **Duration of research:** At this time your participation will last about 10–15 min to complete the survey. Based on your responses to this survey, you may be invited to participate in a follow-up study.
4. **Study procedures:** The survey consists of questions dealing with your thoughts about health care for culturally and ethnically diverse patients. There are also a few questions about your broader values and beliefs about the world.
5. **Possible risks and discomfort:** Apart from the risk to confidentiality discussed below, there is the possibility that you may be uncomfortable answering some of the questions. You are fee to skip any questions you wish not to answer, or you may withdraw from the study at any time by emailing the Principal Investigator (diana.burgess@va.gov).
6. **Potential benefits:** There is no benefit to you for participation in this study.
7. **Indirect benefits**. This research will help guide the development of strategies to improve health care within the VA, particularly for racially and ethnically diverse patients.
8. **Confidentiality:** Your responses will be completely confidential. Your responses will be stored securely and separately from your name and email address, and only researchers and technical support staff with security clearances will have access to the record. A file containing your identifying information is stored on a secure, password protected server within the VA and will only be used to send e-mails inviting your participation in this study and a potential follow-up study. We will report study findings aggregately and will never report on a specific participant or a specific VA facility.
9. Participation is voluntary and your decision not to take part will not affect your employment.
10. There are no costs to you for participating and you will receive no payment for participation.
11. **Persons to contact about this study.** You can contact the Principal Investigator, Dr Diana Burgess (diana.burgess@va.gov; phone: 612-467-1591) with any questions, complaints, and concerns about the research or related matters. You can also contact another Co-Investigator Dr. Melissa Partin; Minneapolis VAHCS, Melissa.partin@va.gov; (612) 467-3841.
I f you have questions about your rights as a study participant, or you want to make sure this is a valid VA study, you may contact the VA Central Institutional Review Board (IRB). This is the Board that is responsible for overseeing the safety of human participants in this study. You may call the VA Central IRB toll free at 1-877-254-3130 if you have questions, complaints, or concerns about the study or if you would like to obtain information or offer input.
12. **Agreement to participate in research**: Please click on the link http://vhaminweb3/ccdordev/empowerproject/surveyindex.asp stating that (1) you have read the information above, (2) if you have questions you understand whom to contact, and (3) you voluntarily agree to participate in this study.

## References

[1] Institute of Medicine (US) Committee on Understanding and eliminating racial and ethnic disparities in health care. In: Unequal Treatment: Confronting Racial and Ethnic Disparities in Health Care. Edited by Smedley BD, Stith AY, Nelson AR Washington, DC: National Academies Press (US), 2003 25032386

[2] Cook BL, McGuire T, Miranda J Measuring trends in mental health care disparities, 2000–2004. Psychiatr Serv. 2007;58:1533–1540 18048553 10.1176/ps.2007.58.12.1533

[3] Jimenez DE, Cook B, Bartels SJ, et al. Disparities in mental health service use of racial and ethnic minority elderly adults. J Am Geriatr Soc. 2013;61:18–25 23252464 10.1111/jgs.12063 PMC3545089

[4] Saha S, Freeman M, Toure J, et al. Racial and ethnic disparities in the VA health care system: a systematic review. J Gen Intern Med. 2008;23:654–671 18301951 10.1007/s11606-008-0521-4 PMC2324157

[5] Biener AI, Zuvekas SH Do racial and ethnic disparities in health care use vary with health? Health Serv Res. 2019;54:64–74 30430571 10.1111/1475-6773.13087 PMC6338306

[6] Alexander GC, Lin S, Sayla MA, et al. Development of a measure of physician engagement in addressing racial and ethnic health care disparities. Health Serv Res. 2008;43:773–784 18370978 10.1111/j.1475-6773.2007.00780.x PMC2442375

[7] Koh HK, Graham G, Glied SA Reducing racial and ethnic disparities: the action plan from the department of health and human services. Health Aff. 2011;30:1822–1829 10.1377/hlthaff.2011.0673 21976322

[8] Exworthy M, Morcillo V Primary care doctors' understandings of and strategies to tackle health inequalities: a qualitative study. Prim Health Care Res Dev. 2019;20:1–7 10.1017/S146342361800052X PMC6536748 32800013

[9] Kano M, Sanchez N, Tami-Maury I, et al. Addressing cancer disparities in SGM populations: recommendations for a national action plan to increase SGM health equity through researcher and provider training and education. J Cancer Educ. 2018 DOI:10.1007/s13187-018-1438-1 PMC10368403 30377952

[10] Butler M, McCreedy E, Schwer N, et al. Comparative Effectiveness Review No. 170. In: Improving Cultural Competence to Reduce Health Disparities. AHRQ Publication No. 16-EHC006-EF. Rockville, MD: Agency for Healthcare Research and Quality (US), 2016 27148614

[11] Nelson SC, Prasad S, Hackman HW Training providers on issues of race and racism improve health care equity. Pediatr Blood Cancer. 2015;62:915–917 25683782 10.1002/pbc.25448

[12] Horvat L, Horey D, Romios P, et al. Cultural competence education for health professionals. Cochrane Database Syst Rev. 2014:Cd009405 24793445 10.1002/14651858.CD009405.pub2 PMC10680054

[13] Lie DA, Lee-Rey E, Gomez A, et al. Does cultural competency training of health professionals improve patient outcomes? A systematic review and proposed algorithm for future research. J Gen Intern Med. 2011;26:317–325 20953728 10.1007/s11606-010-1529-0 PMC3043186

[14] Shepherd SM Cultural awareness workshops: limitations and practical consequences. BMC Med Educ. 2019;19:14 30621665 10.1186/s12909-018-1450-5 PMC6325797

[15] Burgess DJ, Bokhour BG, Cunningham BA, et al. Communicating with providers about racial healthcare disparities: the role of providers' prior beliefs on their receptivity to different narrative frames. Patient Educ Couns. 2019;102:139–147 30266266 10.1016/j.pec.2018.08.030

[16] Burgess DJ, Bokhour BG, Cunningham BA, et al. Healthcare providers' responses to narrative communication about racial healthcare disparities. Health Commun. 2019;34:149–161 29068701 10.1080/10410236.2017.1389049

[17] Nelson TE, Garst J Values-based political messages and persuasion: relationships among speaker, recipient, and evoked values. Polit Psychol. 2005;26:489–516

[18] Byrne S, Hart PS The boomerang effect a synthesis of findings and a preliminary theoretical framework. Ann Int Commun Assoc. 2009;33:3–37

[19] Britton BV, Nagarajan N, Zogg CK, et al. us surgeons' perceptions of racial/ethnic disparities in health care: a cross-sectional study. JAMA Surg. 2016;151:582–584 26818695 10.1001/jamasurg.2015.4901

[20] Greysen SR, Siegel B, Sears V, et al. Residents' awareness of racial and ethnic disparities in cardiovascular care. J Graduate Med Educ. 2011;3:417–420 10.4300/JGME-D-10-00190.1 PMC3179245 22942977

[21] Kendrick J, Nuccio E, Leiferman JA, et al. Primary care providers perceptions of racial/ethnic and socioeconomic disparities in hypertension control. Am J Hypertens. 2015;28:1091–1097 25631381 10.1093/ajh/hpu294 PMC4542848

[22] Lurie N, Fremont A, Jain AK, et al. Racial and ethnic disparities in care: the perspectives of cardiologists. Circulation. 2005;111:1264–1269 15769767 10.1161/01.CIR.0000157738.12783.71

[23] Sequist TD, Ayanian JZ, Marshall R, et al. Primary-care clinician perceptions of racial disparities in diabetes care. J Gen Intern Med. 2008;23:678–684 18214625 10.1007/s11606-008-0510-7 PMC2324133

[24] Nelson A Unequal treatment: confronting racial and ethnic disparities in health care. J Natl Med Assoc. 2002;94:666 12152921 PMC2594273

[25] Taylor SL, Fremont A, Jain AK, et al. Racial and ethnic disparities in care: the perspectives of cardiovascular surgeons. Ann Thorac Surg. 2006;81:531–536 16427845 10.1016/j.athoracsur.2005.08.004

[26] Gollust SE, Cunningham BA, Bokhour BG, et al. What causes racial health care disparities? A mixed-methods study reveals variability in how health care providers perceive causal attributions. Inquiry. 2018;55:46958018762840 29553296 10.1177/0046958018762840 PMC5862368

[27] Padela AI, Punekar IR Emergency medical practice: advancing cultural competence and reducing health care disparities. Acad Emerg Med. 2009;16:69–75 19055674 10.1111/j.1553-2712.2008.00305.x

[28] Nesbitt S, Palomarez RE Review: increasing awareness and education on health disparities for health care providers. Ethn Dis. 2016;26:181–190 27103768 10.18865/ed.26.2.181 PMC4836898

[29] Statistics NCfVAa. 2014 Minority Veterans Report. 2016. Available at https://www.va.gov/vetdata/docs/SpecialReports/Minority_Veterans_2014.pdf Accessed 215, 2019

[30] VHA. Veterans Health Administration. 2017. Available at https://www.va.gov/health/aboutvha.asp Accessed 215, 2019

[31] Uchendu US. Institutional journey in pursuit of health equity: veterans Health Administration's Office of Health Equity. Am J Public Health. 2014;104:S511–S513 25100409 10.2105/AJPH.2014.302183 PMC4151896

[32] VHA. US Department of Veterans Affairs Office of Health Equity Mission and Accomplishments. 2015 Available at https://www.va.gov/HEALTHEQUITY/docs/OHE_Mission_and_Accomplishments_November_2015.pdf Accessed 215, 2019

[33] Chernick MR Bootstrap Methods: A Guide for Practitioners and Researchers. Vol. 619 John Wiley & Sons, 2011

[34] Valentino NA, Sears DO Old times there are not forgotten: race and partisan realignment in the contemporary South. Am J Pol Sci. 2005;49:672–688

[35] Kuklinski JH, Cobb MD, Gilens M Racial attitudes and the “New South”. J Politics. 1997;59:323–349

[36] Li S, Chen A, Mead K Racial disparities in the use of cardiac revascularization: does local hospital capacity matter? PLoS One. 2013;8:e69855 23875005 10.1371/journal.pone.0069855 PMC3713060

[37] Varkey AB, Manwell LB, Williams ES, et al. Separate and unequal: clinics where minority and nonminority patients receive primary care. Arch Intern Med. 2009;169:243–250 19204215 10.1001/archinternmed.2008.559 PMC13012804

[38] Reschovsky JD, O'Malley AS Do primary care physicians treating minority patients report problems delivering high-quality care? Health Aff. 2008;27:w222–w231 10.1377/hlthaff.27.3.w222 18430747

[39] Burgess DJ Are providers more likely to contribute to healthcare disparities under high levels of cognitive load? How features of the healthcare setting may lead to biases in medical decision making. Med Decis Making. 2010;30:246–257 19726783 10.1177/0272989X09341751 PMC3988900

[40] Malat J The appeal and problems of a cultural competence approach to reducing racial disparities. J Gen Intern Med. 2013;28:605–607 23404202 10.1007/s11606-013-2363-y PMC3631069

[41] Feagin J, Bennefield Z Systemic racism and U.S. health care. Soc Sci Med. 2014;103:7–14 24507906 10.1016/j.socscimed.2013.09.006

[42] Blane D Social determinants of health—socioeconomic status, social class, and ethnicity. Am J Public Health. 1995;85:903–905 7604907 10.2105/ajph.85.7.903 PMC1615518

[43] US Department of Health and Human Services. Healthy People 2020. 2010. Available at www.healthypeople.gov/2020/about/foundation-health-measures/Determinants-of-Health Accessed 718, 2019

[44] Baum FE, Begin M, Houweling TA, et al. Changes not for the fainthearted: reorienting health care systems toward health equity through action on the social determinants of health. Am J Public Health. 2009;99:1967–1974 19762660 10.2105/AJPH.2008.154856 PMC2759791

[45] Lewis FM, Daltroy LH How Causal Explanations Influence Health Behavior: Attribution Theory. San Francisco, CA: Jossey-Bass, 1990

[46] Price JH, Braun RE, Khubchandani J, et al. Development of an attribution of racial/ethnic health disparities scale. J Community Health. 2014;39:792–799 24499967 10.1007/s10900-014-9833-y

[47] Watt RG From victim blaming to upstream action: tackling the social determinants of oral health inequalities. Community Dent Oral Epidemiol. 2007;35:1–11 17244132 10.1111/j.1600-0528.2007.00348.x

[48] Braveman P, Gottlieb L The social determinants of health: it's time to consider the causes of the causes. Public Health Rep. 2014;129 Suppl 2:19–31 10.1177/00333549141291S206 PMC3863696 24385661

[49] Gkiouleka A, Huijts T, Beckfield J, et al. Understanding the micro and macro politics of health: inequalities, intersectionality & institutions—a research agenda. Soc Sci Med. 2018;200:92–98 29421476 10.1016/j.socscimed.2018.01.025

[50] Short SE, Mollborn S Social determinants and health behaviors: conceptual frames and empirical advances. Curr Opin Psychol. 2015;5:78–84 26213711 10.1016/j.copsyc.2015.05.002 PMC4511598

[51] Veterans Health Administration. Whole Health For Life. 2018. Available at https://www.va.gov/patientcenteredcare Accessed 718, 2019

[52] Betancourt JR Cross-cultural medical education: conceptual approaches and frameworks for evaluation. Acad Med. 2003;78:560–569 12805034 10.1097/00001888-200306000-00004

[53] Cipriano GC, Andrews CO The Hispanic pharmacist: value beyond a common language. SAGE Open Med. 2015;3:2050312115581250 26770782 10.1177/2050312115581250 PMC4679233

[54] Cohen JJ, Gabriel BA, Terrell C The case for diversity in the health care workforce. Health Aff. 2002;21:90–102 10.1377/hlthaff.21.5.90 12224912

[55] Jackson CS, Gracia JN Addressing health and health-care disparities: the role of a diverse workforce and the social determinants of health. Public Health Rep. 2014;129 Suppl 2:57–61 24385666 10.1177/00333549141291S211 PMC3863703

[56] Reede JY A recurring theme: the need for minority physicians. Health Aff. 2003;22:91–93 10.1377/hlthaff.22.4.91 12889755

[57] Ashton CM, Haidet P, Paterniti DA, et al. Racial and ethnic disparities in the use of health services: bias, preferences, or poor communication? J Gen Intern Med. 2003;18:146–152 12542590 10.1046/j.1525-1497.2003.20532.x PMC1494820

[58] Charles C, Gafni A, Whelan T Shared decision-making in the medical encounter: what does it mean? (or it takes at least two to tango). Soc Sci Med. 1997;44:681–692 9032835 10.1016/s0277-9536(96)00221-3

[59] Eliacin J, Salyers MP, Kukla M, et al. Factors influencing patients' preferences and perceived involvement in shared decision-making in mental health care. J Ment Health. 2015;24:24–28 25279691 10.3109/09638237.2014.954695 PMC9520587

[60] Eliacin J, Salyers MP, Kukla M, Matthias MS Patients' understanding of shared decision making in a mental health setting. Qual Health Res. 2015;25:668–678 25246333 10.1177/1049732314551060

[61] Whitley R The implications of race and ethnicity for shared decision-making. Psychiatr Rehabil J. 2009;32:227–230 19136356 10.2975/32.3.2009.227.230

[62] Atkins D, Kilbourne A, Lipson L Health equity research in the Veterans Health Administration: we've come far but aren't there yet. Am J Public Health. 2014;104 Suppl 4:S525–S526 25100414 10.2105/AJPH.2014.302216 PMC4151887

[63] Trivedi AN, Grebla RC, Wright SM, et al. Despite improved quality of care in the Veterans Affairs health system, racial disparity persists for important clinical outcomes. Health Aff. 2011;30:707–715 10.1377/hlthaff.2011.0074 21471492

